# Supplementary material for: Post-Diagnostic Support for Behaviour Changes in Young-Onset Dementia in Australia
Source: Brain Sci. 2023 Oct 30;13(11):1529. doi: 10.3390/brainsci13111529 (PMC10670083; doi:10.3390/brainsci13111529)
Supplement: Supplementary file 1 [file brainsci-13-01529-s001.zip › brainsci-2672521-supplementary.pdf]

Supplementary material: Post-diagnostic support for behaviour changes in young-onset dementia in Australia

Claire J. Cadwallader, Dennis Velakoulis, Samantha M. Loi

Contents

Supplementary methods (pp. 2-15)

Questionnaire items

Supplementary results (pp. 16)

GP support satisfaction ratings (Figure S1).

Emergency support satisfaction ratings (Figure S2).

Private specialist satisfaction ratings (Figure S3)

Adult mental health service satisfaction ratings (Figure S4)

Older adult mental health service satisfaction ratings (Figure S5)

Dementia Services Australia satisfaction ratings (Figure S6)

Supplementary methods

Table S1. Questionnaire items

The following survey was designed within the platform RedCAP and includes branching logic, such that the questions participants answer depend on their responses to preceding questions. The following copy of the survey displays all possible questions and does not show the branching logic. The bullet points below explain the main functions of the branching logic:

- Participants with younger-onset dementia (YOD) were not required to complete the Neuropsychiatric Inventory Questionnaire (NPI-Q) as this is a proxy measure designed to be filled out by caregivers.
- Healthcare practitioners and paid support workers/coordinators working for an organisation that supports people with YOD were not required to answer questions about specific details of a YOD diagnosis or the NPI-Q as they were likely to be working with multiple people who have YOD. They were still asked about the specific types of behaviour changes they needed support for.
- In the ‘Satisfaction’ section of the survey, participants were asked to rate their satisfaction for only those services that they selected as having provided them with support for the behaviour changes.

Are you a person who is living with younger-onset dementia (a dementia with symptom onset at < 65 years old)?	◯ Yes<br>◯ No
Are you (main role):	◯ A family carer/support person (unpaid/informal) living with someone who has younger-onset dementia (YOD)<br>◯ Healthcare practitioner (paid/formal) working in the community who looks after a person with younger-onset dementia (YOD)<br>◯ Healthcare practitioner (paid/formal) working in a residential care facility who looks after someone with younger-onset dementia (YOD)<br>◯ A paid carer working in the community who looks after a person with younger-onset dementia (YOD)<br>◯ A paid carer working in a residential care facility who looks after a person with younger-onset dementia (YOD)<br>◯ A paid support worker/coordinator working for an organisation (e.g Dementia Australia, Huntington's Vic etc) that looks after someone with younger-onset dementia (YOD)
Is your professional role	◯ Medical<br>◯ Nursing<br>◯ Allied health<br>◯ Personal care attendant<br>◯ Other
Is your role supporting a person with YOD part of the National Disability Insurance Scheme (NDIS)?	◯ Yes<br>◯ No<br>◯ Maybe/Not sure
Do you have a package by the National Disability Insurance Scheme (NDIS)?	◯ Yes<br>◯ No<br>◯ We/I have made an NDIS access request and I'm awaiting my planning meeting<br>◯ Our/my NDIS application was rejected<br>◯ We/I have an aged care package<br>◯ I don't know
What is your current age?	________________________________
What is your gender?	◯ Identify as a woman<br>◯ Identify as a man<br>◯ Identify as non-binary<br>◯ Prefer not to say
Which state or territory do you live	◯ Vic<br>◯ NSW<br>◯ Queensland<br>◯ South Australia<br>◯ Western Australia<br>◯ ACT<br>◯ Tasmania<br>◯ Northern Territory
What is your living situation	◯ Live alone<br>◯ Live with family/partner<br>◯ Live in shared accommodation<br>◯ Live in residential care facility<br>◯ Other

Do you live with the person with younger-onset dementia who you are caring for?	◯ Yes<br>◯ No - they live in a residential care facility<br>◯ No - they live in Specialist Disability Accommodation, SDA (specialist designed housing for people with high intensity needs)<br>◯ No - they live in Supported Independent Living, SIL (housing with onsite supports and daily living services for support)<br>◯ No - I am unsure whether they live in SDA nor SIL but we do not live together
Is where you live	◯ Metropolitan/urban<br>◯ Rural<br>◯ Remote<br>◯ Not sure
Is the residential care facility you work at located in	◯ Metropolitan/urban<br>◯ Rural<br>◯ Remote<br>◯ Not sure
What is your postcode	________________________
What is the postcode of where you work	________________________

Details of younger-onset dementia

Please complete the survey below.

Thank you!

What is the type of younger-onset dementia?	☐ Alzheimer's<br>☐ Posterior cortical atrophy<br>☐ Frontotemporal dementia<br>☐ Language variant dementia<br>☐ Behavioural-variant dementia<br>☐ Frontotemporal dementia with motor neuron disease<br>☐ Vascular dementia<br>☐ Alcohol-related dementia<br>☐ Huntington's disease<br>☐ Dementia with Lewy bodies<br>☐ Other<br>☐ Not sure
If you answered 'Other', what is the type of younger-onset dementia?	______________________________
If you know, what was the age of onset of the younger-onset dementia, (i.e when did you first notice symptoms that could be a dementia)?	________________________
If you know, what was the age of diagnosis of the younger-onset dementia?	________________________
If you know, when was the diagnosis given? (Month/Year)	________________________
If you know, who gave the diagnosis of younger-onset dementia?	☐ GP<br>☐ Psychiatrist<br>☐ Neurologist<br>☐ Geriatrician<br>☐ Neuropsychologist<br>☐ Nursing<br>☐ Allied health<br>☐ Other<br>☐ Don't know

Have you noticed or has anyone else noticed, any behaviour changes since the onset of dementia, which you would like advice or support for?	◯ Yes<br>◯ No
What types of behaviour changes associated with younger-onset dementia have you wanted support for?	☐ False beliefs such as thinking others are stealing things or planning to harm (delusions)<br>☐ False visions or voices /seeing or hearing things that aren't there (hallucinations)<br>☐ Resistive to help from others or hard to handle (agitation / aggression)<br>☐ Feel/appear sad or depressed (depression)<br>☐ Feel/appear upset when separated or nervous (anxiety)<br>☐ Feel/appear too good or excessively happy (elation / euphoria)<br>☐ Less interested in plans or usual activities (apathy / indifference)<br>☐ Act impulsively or say things that might hurt other's feelings (disinhibition)<br>☐ Act impatiently or cranky, difficulty coping with delays or waiting (Irritability /lability)<br>☐ Have repetitive behaviours such as pacing, handling buttons or doing other things repeatedly (motor disturbance)<br>☐ Wake during the night, get up too early in the morning or have excessive naps during the day (night time behaviours)<br>☐ Have lost or gained weight or had a change in food preferences (appetite /eating changes)<br>☐ Other
Have you sought help or support for the behaviour changes?	◯ Yes<br>◯ No<br>◯ Not sure

NPI-Q and support received

Please complete the survey below.

Thank you!

Were any of these behaviour changes present in the last month?	☐ False beliefs such as thinking others are stealing things or planning to harm them (delusions)<br>☐ False visions or voices /seeing or hearing things that aren't there (hallucinations)<br>☐ Resistive to help from others or hard to handle (agitation / aggression)<br>☐ Appear sad or say they are depressed (depression)<br>☐ Appear upset when separated or nervous (anxiety)<br>☐ Appear too good or excessively happy (elation / euphoria)<br>☐ Less interested in plans or usual activities (apathy / indifference)<br>☐ Act impulsively or say things that might hurt other's feelings (disinhibition)<br>☐ Act impatiently or cranky, difficulty coping with delays or waiting (Irritability /lability)<br>☐ Have repetitive behaviours such as pacing, handling buttons or doing other things repeatedly (motor disturbance)<br>☐ Wake during the night, get up too early in the morning or have excessive naps during the day (night time behaviours)<br>☐ Have lost or gained weight or had a change in the food they like (appetite /eating changes)<br>☐ Other
What is the severity of these delusions for the PERSON WITH YOUNGER-ONSET DEMENTIA?	◯ Mild - noticeable but not a significant change<br>◯ Moderate - significant but not a dramatic change<br>◯ Severe - marked or prominent change<br>◯ No change<br>◯ Don't know

How distressing do YOU find the delusions?	◯ Not distressing at all<br>◯ Minimal - slightly distressing, not a problem to cope with<br>◯ Mild - not very distressing, generally easy to cope with<br>◯ Moderate - fairly distressing, not always easy to cope with<br>◯ Severe - very distressing, difficult to cope with<br>◯ Very severe - extremely distressing, unable to cope with
What is the severity of these hallucinations for the PERSON WITH YOUNGER-ONSET DEMENTIA?	◯ Mild - noticeable but not a significant change<br>◯ Moderate - significant but not a dramatic change<br>◯ Severe - marked or prominent change<br>◯ No change<br>◯ Don't know
How distressing do YOU find the hallucinations?	◯ Not distressing at all<br>◯ Minimal - slightly distressing, not a problem to cope with<br>◯ Mild - not very distressing, generally easy to cope with<br>◯ Moderate - fairly distressing, not always easy to cope with<br>◯ Severe - very distressing, difficult to cope with<br>◯ Very severe - extremely distressing, unable to cope with
What is the severity of the agitation/aggression for the PERSON WITH YOUNGER-ONSET DEMENTIA?	◯ Mild - noticeable but not a significant change<br>◯ Moderate - significant but not a dramatic change<br>◯ Severe - marked or prominent change<br>◯ No change<br>◯ Don't know
How distressing do YOU find the agitation/aggression?	◯ Not distressing at all<br>◯ Minimal - slightly distressing, not a problem to cope with<br>◯ Mild - not very distressing, generally easy to cope with<br>◯ Moderate - fairly distressing, not always easy to cope with<br>◯ Severe - very distressing, difficult to cope with<br>◯ Very severe - extremely distressing, unable to cope with
What is the severity of depressed mood for the PERSON WITH YOUNGER-ONSET DEMENTIA?	☐ Mild - noticeable but not a significant change<br>☐ Moderate - significant but not a dramatic change<br>☐ Severe - marked or prominent change<br>☐ No change<br>☐ Don't know
How distressing for YOU is the depressed mood?	☐ Not distressing at all<br>☐ Minimal - slightly distressing, not a problem to cope with<br>☐ Mild - not very distressing, generally easy to cope with<br>☐ Moderate - fairly distressing, not always easy to cope with<br>☐ Severe - very distressing, difficult to cope with<br>☐ Very severe - extremely distressing, unable to cope with
What is the severity of the anxiety for the PERSON WITH YOUNGER-ONSET DEMENTIA?	◯ Mild - noticeable but not a significant change<br>◯ Moderate - significant but not a dramatic change<br>◯ Severe - marked or prominent change<br>◯ No change<br>◯ Don't know

How distressing for YOU is the anxiety?	◯ Not distressing at all<br>◯ Minimal - slightly distressing, not a problem to cope with<br>◯ Mild - not very distressing, generally easy to cope with<br>◯ Moderate - fairly distressing, not always easy to cope with<br>◯ Severe - very distressing, difficult to cope with<br>◯ Very severe - extremely distressing, unable to cope with
What is the severity of the elation/euphoria for the PERSON WITH YOUNGER-ONSET DEMENTIA?	◯ Mild - noticeable but not a significant change<br>◯ Moderate - significant but not a dramatic change<br>◯ Severe - marked or prominent change<br>◯ No change<br>◯ Don't know
How distressing for YOU is the elation/euphoria?	◯ Not distressing at all<br>◯ Minimal - slightly distressing, not a problem to cope with<br>◯ Mild - not very distressing, generally easy to cope with<br>◯ Moderate - fairly distressing, not always easy to cope with<br>◯ Severe - very distressing, difficult to cope with<br>◯ Very severe - extremely distressing, unable to cope with
What is the severity of the apathy/indifference for the PERSON WITH YOUNGER-ONSET DEMENTIA?	◯ Mild - noticeable but not a significant change<br>◯ Moderate - significant but not a dramatic change<br>◯ Severe - marked or prominent change<br>◯ No change<br>◯ Don't know
How distressing for YOU is the apathy/indifference?	◯ Not distressing at all<br>◯ Minimal - slightly distressing, not a problem to cope with<br>◯ Mild - not very distressing, generally easy to cope with<br>◯ Moderate - fairly distressing, not always easy to cope with<br>◯ Severe - very distressing, difficult to cope with<br>◯ Very severe - extremely distressing, unable to cope with
What is the severity of the disinhibition for the PERSON WITH YOUNGER-ONSET DEMENTIA?	◯ Mild - noticeable but not a significant change<br>◯ Moderate - significant but not a dramatic change<br>◯ Severe - marked or prominent change<br>◯ No change<br>◯ Don't know
How distressing for YOU is the disinhibition?	◯ Not distressing at all<br>◯ Minimal - slightly distressing, not a problem to cope with<br>◯ Mild - not very distressing, generally easy to cope with<br>◯ Moderate - fairly distressing, not always easy to cope with<br>◯ Severe - very distressing, difficult to cope with<br>◯ Very severe - extremely distressing, unable to cope with
What is the severity of the irritability/lability for the PERSON WITH YOUNGER-ONSET DEMENTIA?	◯ Mild - noticeable but not a significant change<br>◯ Moderate - significant but not a dramatic change<br>◯ Severe - marked or prominent change<br>◯ No change<br>◯ Don't know

How distressing for YOU is the irritability/lability?	◯ Not distressing at all<br>◯ Minimal - slightly distressing, not a problem to cope with<br>◯ Mild - not very distressing, generally easy to cope with<br>◯ Moderate - fairly distressing, not always easy to cope with<br>◯ Severe - very distressing, difficult to cope with<br>◯ Very severe - extremely distressing, unable to cope with
What is the severity of the motor disturbance for the PERSON WITH YOUNGER-ONSET DEMENTIA?	◯ Mild - noticeable but not a significant change<br>◯ Moderate - significant but not a dramatic change<br>◯ Severe - marked or prominent change<br>◯ No change<br>◯ Don't know
How distressing for YOU is the motor disturbance?	◯ Not distressing at all<br>◯ Minimal - slightly distressing, not a problem to cope with<br>◯ Mild - not very distressing, generally easy to cope with<br>◯ Moderate - fairly distressing, not always easy to cope with<br>◯ Severe - very distressing, difficult to cope with<br>◯ Very severe - extremely distressing, unable to cope with
What is the severity of the night-time behaviours for the PERSON WITH YOUNGER-ONSET DEMENTIA?	◯ Mild - noticeable but not a significant change<br>◯ Moderate - significant but not a dramatic change<br>◯ Severe - marked or prominent change<br>◯ No change<br>◯ Don't know
How distressing for YOU are the night-time behaviours?	◯ Not distressing at all<br>◯ Minimal - slightly distressing, not a problem to cope with<br>◯ Mild - not very distressing, generally easy to cope with<br>◯ Moderate - fairly distressing, not always easy to cope with<br>◯ Severe - very distressing, difficult to cope with<br>◯ Very severe - extremely distressing, unable to cope with
What is the severity of the appetite/eating changes for the PERSON WITH YOUNGER-ONSET DEMENTIA	◯ Mild - noticeable but not a significant change<br>◯ Moderate - significant but not a dramatic change<br>◯ Severe - marked or prominent change<br>◯ No change<br>◯ Don't know
How distressing for YOU are the appetite/eating changes?	◯ Not distressing at all<br>◯ Minimal - slightly distressing, not a problem to cope with<br>◯ Mild - not very distressing, generally easy to cope with<br>◯ Moderate - fairly distressing, not always easy to cope with<br>◯ Severe - very distressing, difficult to cope with<br>◯ Very severe - extremely distressing, unable to cope with

In the last 12 months, were you able to access help to support you to manage these behaviour changes?	◯ Yes<br>◯ No<br>◯ Not sure
Who did you try to get help for the behaviour changes from?	☐ General practitioner (GP)<br>☐ Went to the Emergency Department<br>☐ Called an ambulance<br>☐ Private specialist (e.g private psychiatrist, private neurologist, private psychologist)<br>☐ Adult mental health team (public)<br>☐ Older adults/aged/older persons mental health team (public)<br>☐ Dementia Australia<br>☐ Dementia Services Australia (DSA)/Dementia Behaviour Management Advisory Services/Severe Behaviour Response Team<br>☐ Other<br>☐ Not sure
Who actually provided you with help for the behaviour changes?	☐ General practitioner (GP)<br>☐ Went to the Emergency Department<br>☐ Called an ambulance<br>☐ Private specialist (e.g private psychiatrist, private neurologist, private psychologist)<br>☐ Adult mental health team (public-funded service for adults, 18-65 years old)<br>☐ Older adults/aged/older persons mental health team (public-funded service for adults aged > 65 years old)<br>☐ Dementia Australia<br>☐ Dementia Services Australia (DSA)/Dementia Behaviour Management Advisory Services/Severe Behaviour Response Team<br>☐ Other<br>☐ Not sure
How long did it take before you were able to get help for these behaviour changes?	◯ Within 1 week<br>◯ 1-2 weeks<br>◯ Within 1 month<br>◯ Within 3 months<br>◯ Within 6 months<br>◯ Longer than 6 months<br>◯ I am still waiting to get support<br>◯ I was declined support from the service/s
Please add here any details of any other support services that you found helpful in providing advice or managing these behaviour changes	________________________________
What form of support did you receive for the behaviour changes?	☐ I spoke to someone on the phone and they gave me advice<br>☐ I had a telehealth/video-conferencing call and they gave me advice<br>☐ I attended an in-person appointment<br>☐ Someone came to do a "home visit" assessment<br>☐ There was an admission to hospital<br>☐ Other
Was the hospital admission	☐ Public adult mental health (adults aged 18-65 years old)<br>☐ Public older adults/aged/older persons mental health (adults aged > 65 years old)<br>☐ Public general hospital<br>☐ Private adult mental health (adults aged 18-65 years old)<br>☐ Private older adults/aged/older persons mental health (adults aged > 65 years old)<br>☐ Private general hospital<br>☐ Other

What were the outcomes of the support you received?	☐ Medication was recommended<br>☐ Non-medication strategies were recommended such as music, distraction etc<br>☐ Both medication and non-medication strategies were recommended<br>☐ Other<br>☐ Not sure
As part of trying to get support for the behaviour changes, have you heard of Dementia Support Australia (DSA)? This includes the Dementia Behaviour Management Advisory Service (DBMAS) and the Severe Behaviour Response Team (SBRT). The DSA is a 24-hour free call service that can provide support and treatment of behaviour changes focusing on non-medication strategies.	◯ Yes<br>◯ No
What kind of contact, if any, have you had with Dementia Services Australia (DSA)?	☐ We made contact and DSA were involved with the care of the person with younger-onset dementia<br>☐ We made contact but DSA did not get involved. We were told to seek support from another organisation<br>☐ We made contact but DSA did not get involved. We were told that we were not appropriate for DSA<br>☐ We made contact and we decided that DSA was not right for us<br>☐ We looked at the DSA website/brochure but did not proceed with contacting them<br>☐ We have heard of DSA through other people but have not had any contact with them
I have not heard of DSA but I might want to contact them in the future for support for the behaviours changes	☐ Yes<br>☐ No<br>☐ Not sure

Page 1

Satisfaction

Please complete the survey below.

Thank you!

Rate how much you agree with the statements

	Completely disagree	Disagree	Neutral/Don't know	Agree	Completely agree	Not applicable
I did not know where to start when looking for a service that was able to help me manage these behaviour changes	◯	◯	◯	◯	◯	◯
It was difficult to access a service which could support me in managing behaviour changes	◯	◯	◯	◯	◯	◯
The process of accessing services to help me manage behaviour changes took too long	◯	◯	◯	◯	◯	◯
It would be useful to have some guidelines for who to contact when you need help for behaviour changes	◯	◯	◯	◯	◯	◯

Which services provided you with support for the behaviour changes associated with younger-onset dementia?	☐ General practitioner (GP)<br>☐ Went to the Emergency Department<br>☐ Called an ambulance<br>☐ Private specialist (e.g private psychiatrist, private neurologist, private psychologist)<br>☐ Adult mental health team (public-funded service for adults 18-65 years old)<br>☐ Older adults/aged/older persons mental health team (public-funded service for adults > 65 years old)<br>☐ Dementia Australia<br>☐ Dementia Services Australia (DSA)/Dementia Behaviour Management Advisory Services/Severe Behaviour Response Team<br>☐ Other<br>☐ I have not needed help

Please rate how much you agree or disagree with the following statements related to the support provided by the Emergency Department

	Completely disagree	Disagree	Neutral/Don't know	Agree	Completely agree	Not applicable
It was easy to access this service	○	○	○	○	○	○
It took too long to access this service	○	○	○	○	○	○
I had a positive experience with this service	○	○	○	○	○	○
I found the service understood younger-onset dementia	○	○	○	○	○	○
I found this service understood the behaviour changes seen in younger-onset dementia	○	○	○	○	○	○
The main outcome from this service was medication for the behaviour changes	○	○	○	○	○	○
The main outcome from this service was use of non-medications e.g., strategies	○	○	○	○	○	○
The main outcome from this service was a combination of medications and strategies	○	○	○	○	○	○
I was overall satisfied with the service	○	○	○	○	○	○
I would use the service again	○	○	○	○	○	○
I would recommend the service to others	○	○	○	○	○	○

Please rate how much you agree or disagree with the following statements related to the support provided by the private specialist

	Completely disagree	Disagree	Neutral/Don't know	Agree	Completely agree	Not applicable
It was easy to access this service	○	○	○	○	○	○
It took too long to access this service	○	○	○	○	○	○
I had a positive experience with this service	○	○	○	○	○	○
I found the service understood younger-onset dementia	○	○	○	○	○	○
I found the service understood the behaviour changes seen in younger-onset dementia	○	○	○	○	○	○
The main outcome from this service was medications for the behaviour changes	○	○	○	○	○	○
The main outcome from this service was use of non-medications e.g., strategies	○	○	○	○	○	○
The main outcome from this service was a combination of medications and strategies	○	○	○	○	○	○
I was overall satisfied with the service	○	○	○	○	○	○
I would use the service again	○	○	○	○	○	○
I would recommend the service to others	○	○	○	○	○	○
I found the service helpful	○	○	○	○	○	○
The service's recommendations resulted in an improvement of the behaviour changes	○	○	○	○	○	○
The service's recommendations helped me feel more confident in managing the behaviour changes	○	○	○	○	○	○

Please rate whether you agree or disagree with the following statements related to the support provided by the adult mental health service (public-funded service for adults 18-65 years old)

	Completely disagree	Disagree	Neutral/Don't know	Agree	Completely agree	Not applicable
It was easy to access this service	○	○	○	○	○	○
It took too long to access this service	○	○	○	○	○	○
I had a positive experience with this service	○	○	○	○	○	○
I found the service understood younger-onset dementia	○	○	○	○	○	○
I found the service understood the behaviour changes seen in younger-onset dementia	○	○	○	○	○	○
The main outcome from this service was medications for the behaviour changes	○	○	○	○	○	○
The main outcome from this service was use of non-medications e.g., strategies	○	○	○	○	○	○
The main outcome from this service for a combination of medications and non-medications e.g., strategies	○	○	○	○	○	○
I was overall satisfied with the service	○	○	○	○	○	○
I would use the service again	○	○	○	○	○	○
I would recommend the service to others	○	○	○	○	○	○
I found the service helpful	○	○	○	○	○	○
The service's recommendations resulted in an improvement in the behaviour changes	○	○	○	○	○	○
The service's recommendations helped me feel more confident in managing the behaviour changes	○	○	○	○	○	○

Please rate whether you agree and disagree with the following statements related to the support provided by the older adults/aged community mental health service (public-funded service for adults > 65 years old)

	Completely disagree	Disagree	Neutral/Don't know	Agree	Completely agree	Not applicable
It was easy to access this service	○	○	○	○	○	○
I had a positive experience with this service	○	○	○	○	○	○
I found the service understood younger-onset dementia	○	○	○	○	○	○
I found the service understood the behaviour changes seen in younger-onset dementia	○	○	○	○	○	○
The main outcome from this service was medications for the behaviour changes	○	○	○	○	○	○
The main outcome from this service is use of non-medications e.g., strategies	○	○	○	○	○	○
The main outcome from this service was a combination of medications and strategies	○	○	○	○	○	○
I was overall satisfied with the service	○	○	○	○	○	○
I would use the service again	○	○	○	○	○	○
I would recommend the service to others	○	○	○	○	○	○
I found the service helpful	○	○	○	○	○	○
The service's recommendations resulted in an improvement in the behaviour changes	○	○	○	○	○	○
The service's recommendations helped me feel more confident in managing the behaviour changes	○	○	○	○	○	○

Please rate whether you agree and disagree with the following statements related to the support provided by Dementia Services Australia.

	Completely disagree	Disagree	Neutral/Don't know	Agree	Completely agree	Not applicable
It was easy to access this service	○	○	○	○	○	○
I had a positive experience with this service	○	○	○	○	○	○
I found the service understood younger-onset dementia	○	○	○	○	○	○
I found the service understood the behaviour changes seen in younger-onset dementia	○	○	○	○	○	○
I found the service helpful	○	○	○	○	○	○
I would use the service again	○	○	○	○	○	○
The service's recommendations resulted in an improvement in the behaviour changes	○	○	○	○	○	○
The service's recommendations helped me feel more confident in managing the behaviour changes	○	○	○	○	○	○
I would recommend the service to others	○	○	○	○	○	○
I was overall satisfied with the service	○	○	○	○	○	○

Please rate whether you agree and disagree with the following statements related to the support provided by your GP

	Completely disagree	Disagree	Neutral/Don't know	Agree	Completely agree	Not applicable
I had a positive experience seeking support from my GP	○	○	○	○	○	○
My GP had a good understanding of younger-onset dementia	○	○	○	○	○	○
My GP knew how to make a referral for me to get more support for the behaviour changes	○	○	○	○	○	○
The service my GP referred me to was appropriate for the support I needed for the behaviour changes	○	○	○	○	○	○

Other comments

Is there anything else you wish to comment on that is related to your experience of seeking or getting support to manage behaviour changes associated with younger-onset dementia? ________________________________

Supplementary results

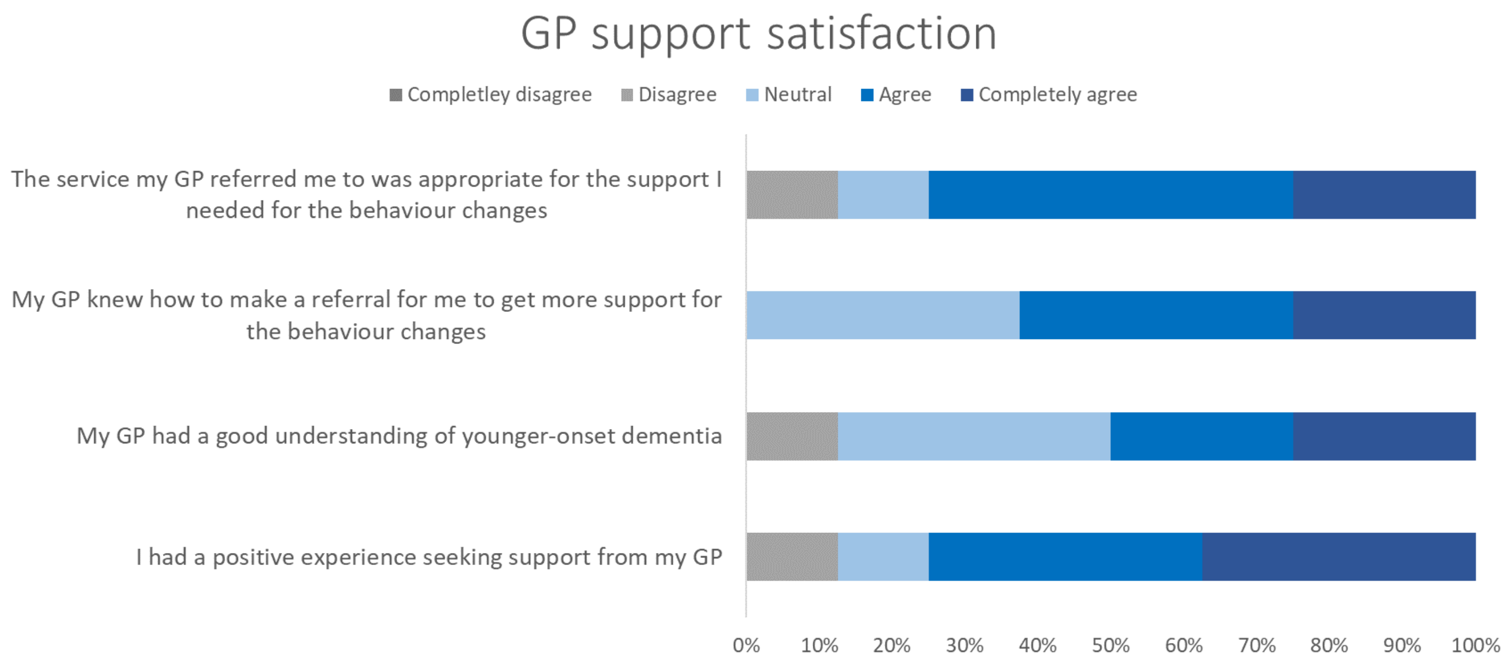

Figure S1. Satisfaction ratings for YOD-related behaviour change support provided by GP’s. N = 8.

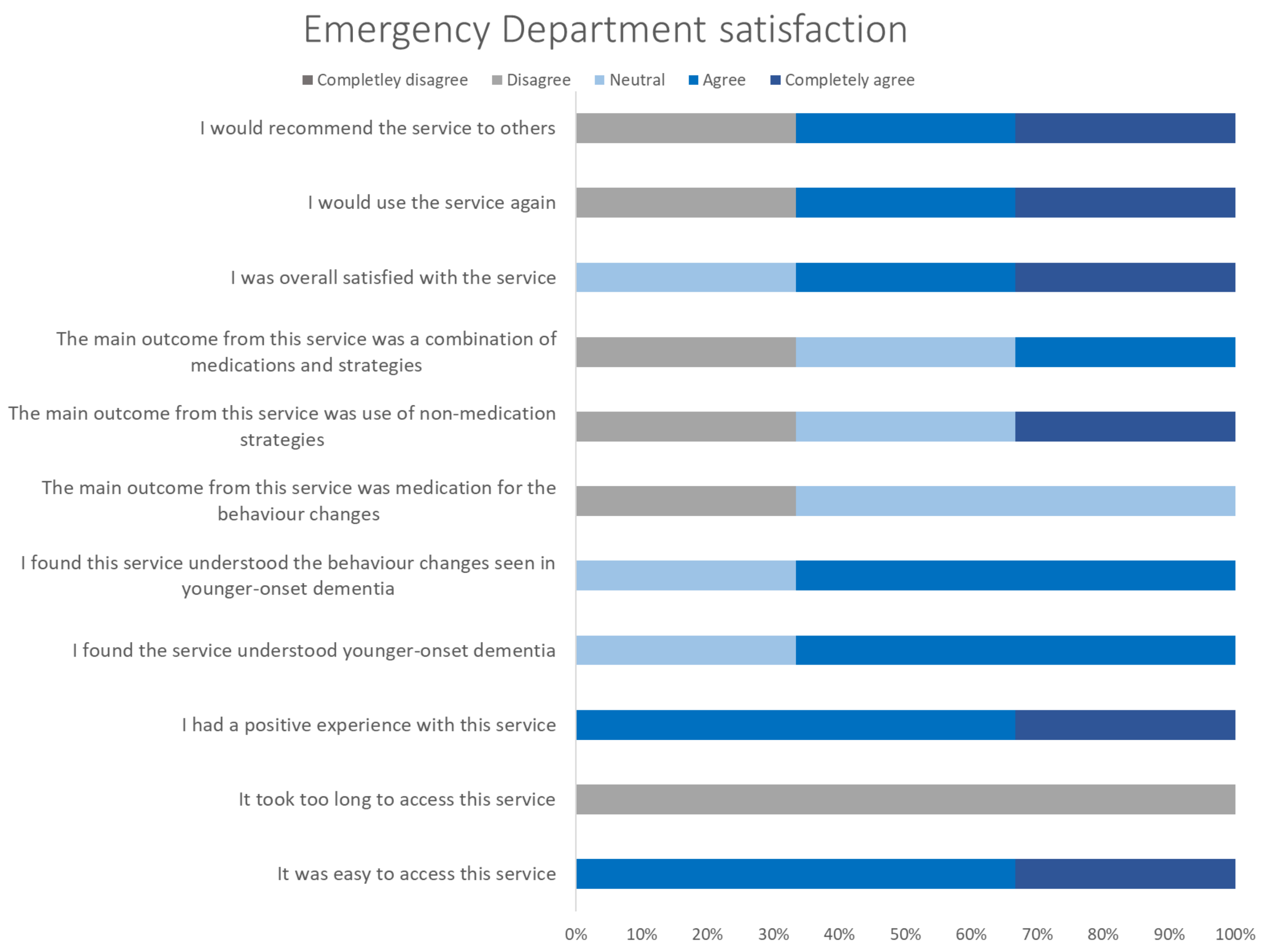

Figure S2. Satisfaction ratings for YOD-related behaviour change support provided by hospital emergency department. N = 3.

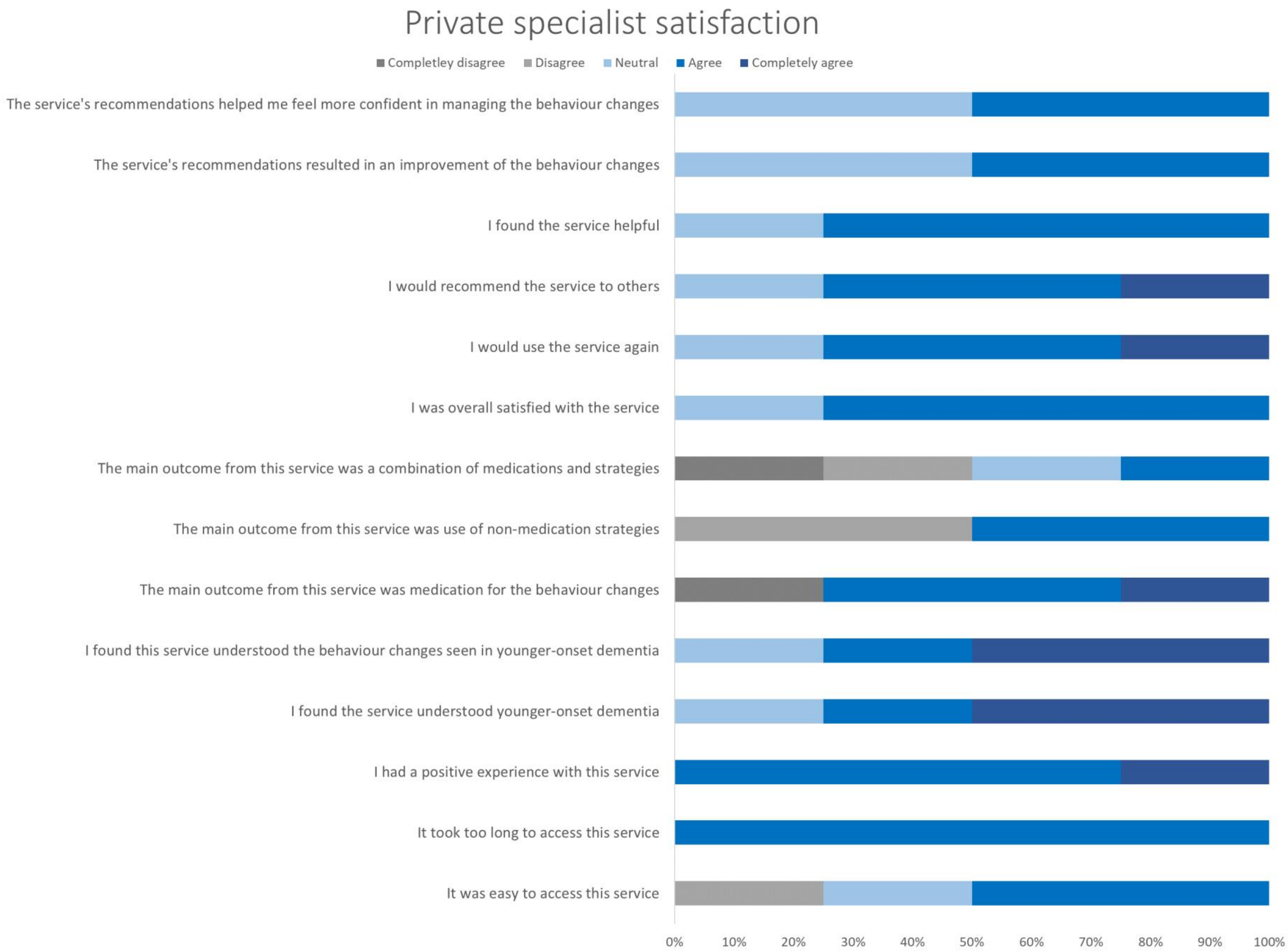

Figure S3. Satisfaction ratings for YOD-related behaviour change support provided by private specialist. N = 4.

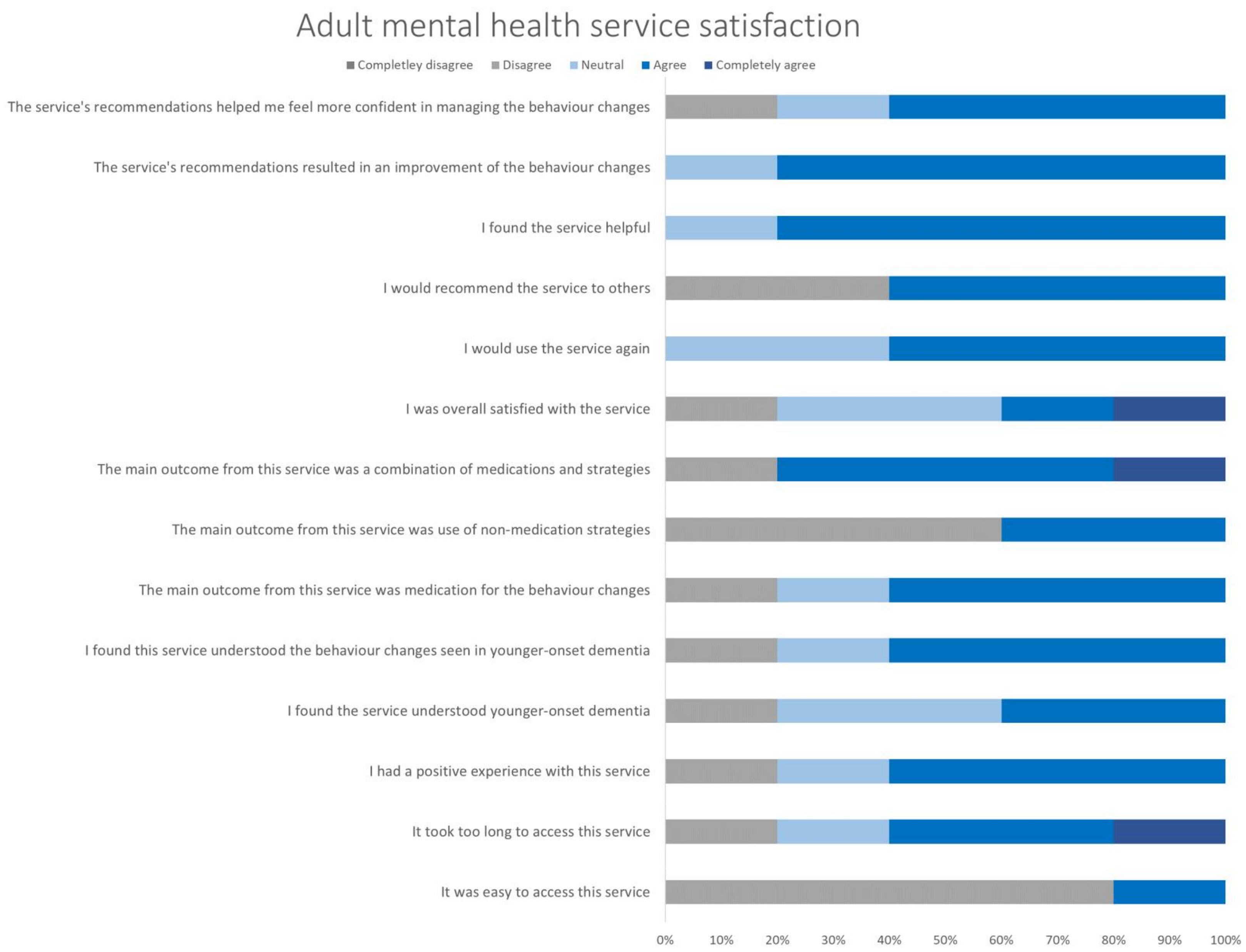

Figure S4. Satisfaction ratings for YOD-related behaviour change support provided by adult mental health service. N = 5.

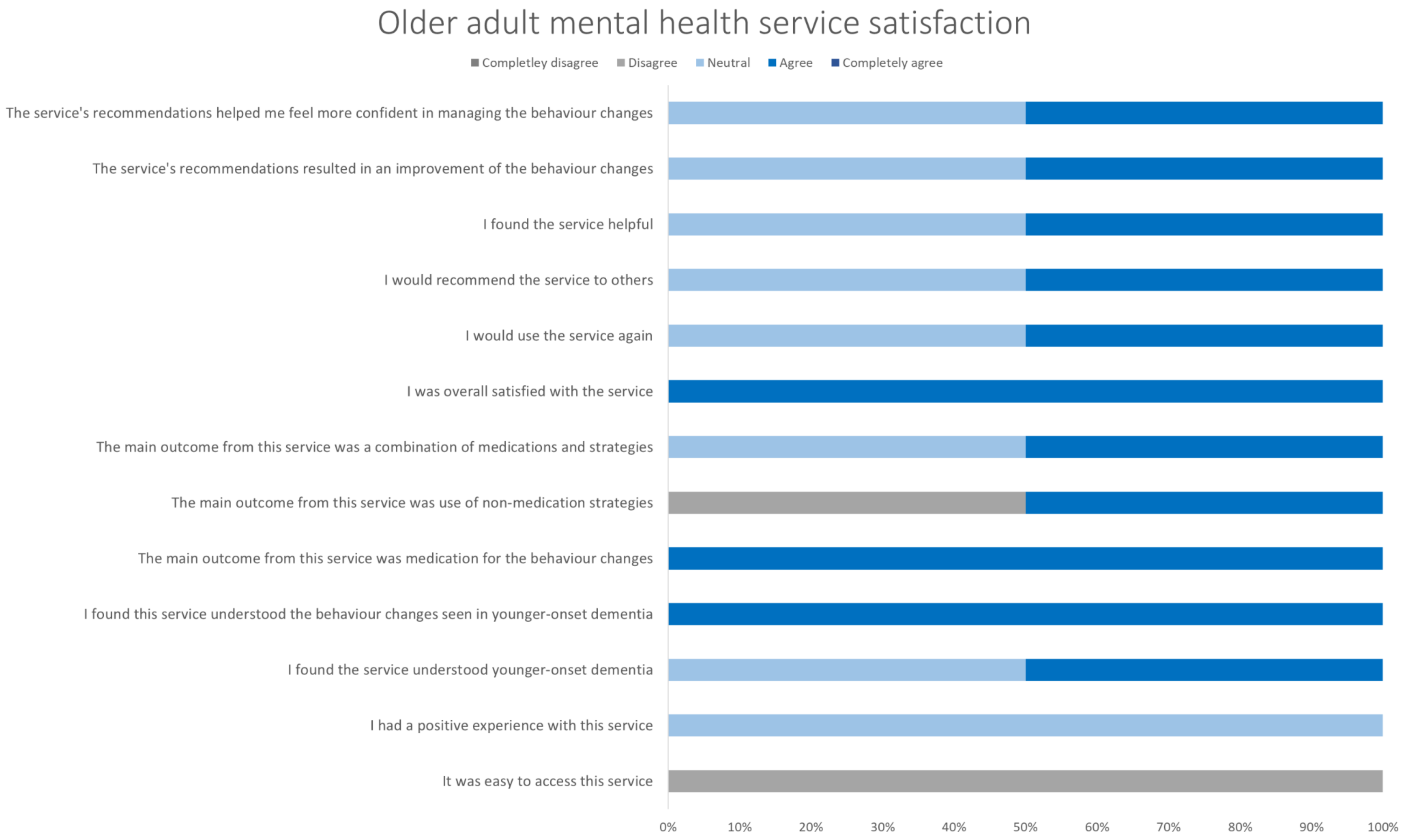

Figure S5. Satisfaction ratings for YOD-related behaviour change support provided by older adult mental health service. N = 2.

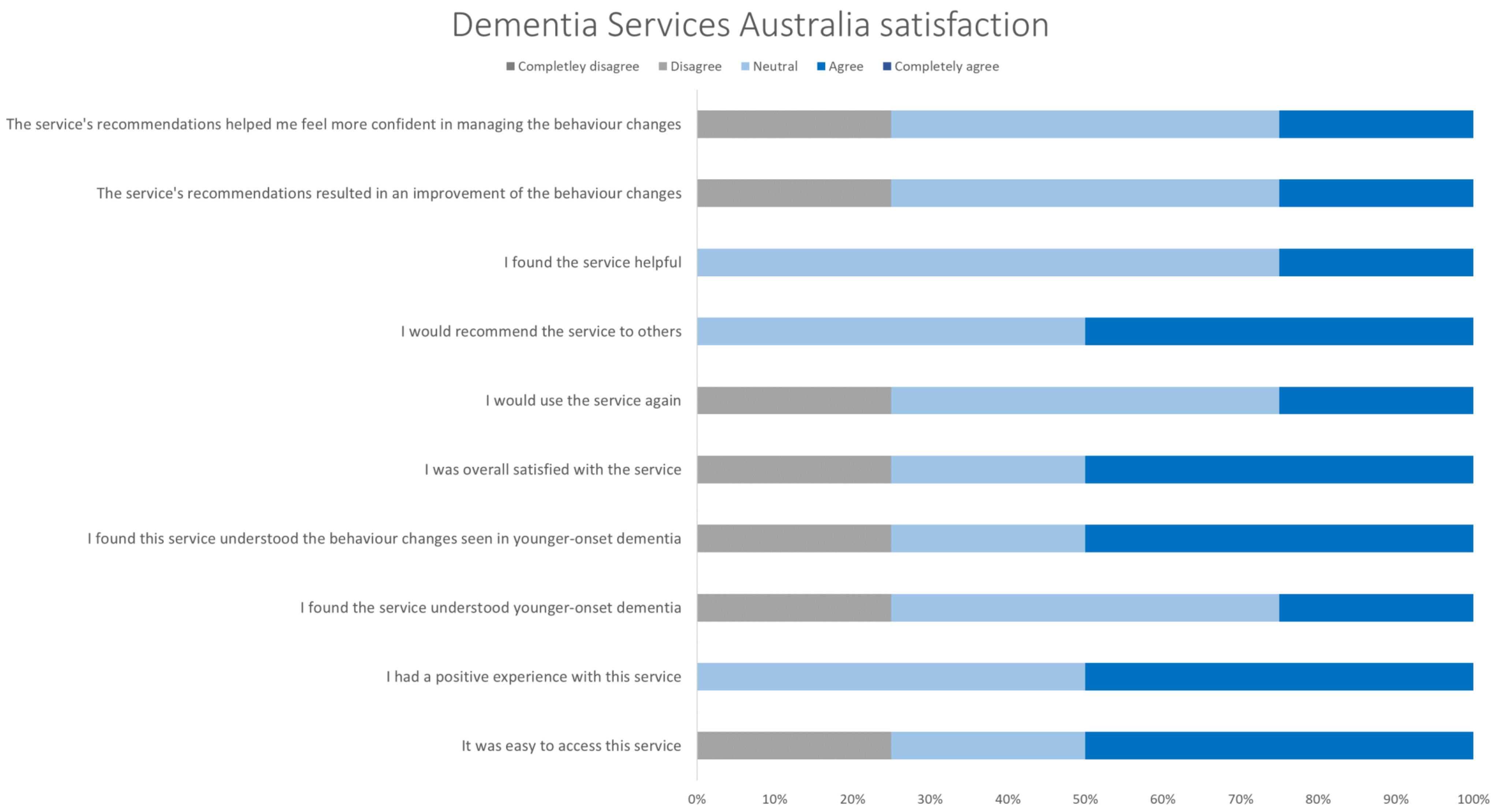

Figure S6. Satisfaction ratings for YOD-related behaviour change support provided by Dementia Services Australia. N = 4.
